# Response of soil microbial community diversity to continuous cucumber cropping in facilities along the Yellow River irrigation area

**DOI:** 10.1371/journal.pone.0289772

**Published:** 2023-08-11

**Authors:** Shuchao Huang, Jihua Yu, Dong Hou, Hongzhong Yue, Dongqin Zhang, Yali Li, Jian Lyu, Li Jin, Ning Jin

**Affiliations:** 1 College of Horticulture, Gansu Agricultural University, Lanzhou, China; 2 Vegetable Research Institute, Gansu Academy of Agricultural Sciences, Lanzhou, China; Sakarya Uygulamali Bilimler Universitesi, TURKEY

## Abstract

Cucumber is an important cash crop; however, continuous cropping obstacles readily occur within the intensive production processes of facility horticulture. This study aimed to determine the effects of continuous cropping on soil quality and the microbial community in the rhizosphere soil of cucumbers. Rhizosphere soil of cucumber planted continuously for 4, 8, and 12 years was investigated, and soil that was not continuously planted was used as the control. Soil physicochemical properties, enzyme activity, microbial diversity, and richness were determined. The results showed that with the increase in continuous cropping years (0, 4, 8, and 12 years), soil total salt content continuously increased, while the pH value significantly decreased. Compared with the control, soil organic matter, alkali-hydrolyzed nitrogen, available phosphorus, available potassium, and nitrate nitrogen contents increased significantly after 4 and 8 years of continuous cropping. Spearman correlation analysis showed that pH was negatively correlated with sucrase or sucrose and available phosphorus was positively correlated with alkaline phosphatase. Compared with the control, the diversity and abundance of bacterial and fungal communities in cucumber rhizosphere soil decreased after 4 and 12 years of continuous cropping. Continuous cropping led to a significant increase in the richness of the dominant phylum of cucumber rhizosphere soil. Principal coordinates analysis showed that, compared with the control, the soil microbial community structure was significantly separated after 4, 8, and 12 years of continuous cropping, and the microbial community structure was most similar after 4 and 8 years of continuous cropping. In addition, redundancy analysis showed that pH was the main driver of soil microbial dominance. In conclusion, continuous cropping of cucumber along the Yellow River irrigation area has led to the deterioration of soil nutrients and microbial communities in that region. This experiment provides a theoretical foundation for addressing the challenges associated with continuous cropping in cucumber cultivation.

## Introduction

Facility agriculture, a typical agricultural production management mode. It plays a crucial role in achieving the annual supply of vegetables and alleviating the food crisis due to its unique production environmental conditions and highly intensive utilization [1]. With the rapid development of facility horticulture in recent years, the area of facility agriculture has expanded annually. Facility agriculture has become a pillar industry to promote agricultural development in all countries of the world, achieve poverty alleviation, and enhance the comprehensive production capacity of agriculture [1, 2]. However, due to cultivated crops in the facilities being limited to single varieties, obstacles related to continuous cropping have become increasingly prominent, especially in the "one village, one product" area of Beiwan Town, Jingyuan County, Gansu Province, China’s dominant production area [3]. For products such as cucumbers [4], peppers [5], and melons [6], applying heavy stubble loads for longer than 10 years has become common. Moreover, continuous cropping has led to the extensive multiplication of soil-borne pathogen [7, 8], as well as the deterioration of ecological conditions, development of serious soil-borne diseases, and destruction of soil nutrient balance, all of which eventually leads to a decline in crop yield and quality, thereby seriously hindering the pace of sustainable development in facility agriculture [9]. Hence, there is an urgent need to identify the primary factors responsible for the challenges in continuous cropping of vegetables and to develop strategies that promote a suitable environment for vegetable growth and development.

Soil microorganisms are one of the most active and diverse components in soil. The composition, activity, and diversity of their communities largely determine the soil nutrient cycling capacity and fertility, which are important biological indicators reflecting soil quality that can be used to evaluate the sustainability of soil ecosystems; moreover, they have an important impact on soil nutrients, structure, and stability [10]. Soil with good stability generally has a strong ability to resist the deterioration of microbial environment, as it has a higher diversity and richness of microorganisms, and is rich in soil nutrients [11]. In soils with serious continuous cropping obstacles, the relative abundance of beneficial microorganisms was found to be low and the relative abundance of harmful microorganisms was high [12, 13]. The study of Gao et al. [14] have confirmed that continuous potato cropping damaged the ecological balance of soil microorganisms and reduced the content of beneficial fungi such as *Chaetomium*, while the content of harmful fungi such as *Verticillium*, *Fusarium*, and *Colletotrichum*, was increased. Similarly, continuous cropping of peanut was found to cause the proliferation of *Fusarium oxysporum*, *Leptosphaerulina australis*, and *Phoma* sp., and increase pathogenic microorganisms such as *Bionectria ochroleuca*, while the relative abundance of beneficial bacteria such as *Trichoderma* sp. and *Mortierella elongata* decreased [15].

In the natural environment, most soil microorganisms are difficult to isolate and cultivate; thus traditional research methods tend to result in obtaining less soil microbial information, which cannot reflect the true community structure or function of soil microorganisms [16]. With the development of high-throughput sequencing technology, the ITS and 16SrDNA methods have been widely used in the study of soil microorganisms in various crops, including tobacco [17], tomato [18], Panax notoginseng [19], and others. ITS and 16SrDNA are used to study all microorganisms within a sample from a specific environment (or habitat), including the community composition, and may be used to interpret the diversity, richness, and population structure of the microbial population, thereby exploring the relationship between microorganisms and the environment or host. Traditional microbial research relies on laboratory culture, and the rise of high-throughput sequencing (e.g., ITS and 16SrDNA) has filled the research gap of being able to study microorganisms that cannot be cultured in traditional laboratories, as well as expanded the use of microbial resources and provided effective tools for studying microbial interactions [20].

Although the effects of facility continuous cropping on soil physicochemical properties, enzyme activities and microorganisms have been widely reported. However, due to the differences in study areas, crop types and planting methods, factors leading to soil degradation in facilities and the change trend of soil microorganisms in continuous cropping period were significantly different [21, 22]. As an important vegetable production base in China, particularly in the northwest, the Yellow River irrigation area in central Gansu Province faces serious continuous cropping obstacles. Specifically, the effects of cucumber continuous cropping on soil quality and soil microorganisms have become increasingly serious, but few studies have investigated this topic. In this study, we investigated the effects of different periods of continuous cropping (0, 4, 8 and 12 years) on the microbial diversity and the community structure of cucumber inter-rhizosphere soils using high-throughput sequencing technology. The objectives of this study were to 1) evaluate the effects of continuous cropping on the physicochemical properties and enzyme activities of inter-rhizosphere soil of cucumber; 2) observe the diversity of inter-rhizosphere soil bacterial and fungal communities of cucumber in response to continuous cropping; and 3) perform the redundancy analysis (RDA) on the dominant soil microbial groups and soil physicochemical factors of cucumber continuous cropping to elucidate the relationships among the continuous cropping and physical and chemical properties of soil factors and soil microorganisms.

## Materials and methods

### Survey of study area and experiment design

Cucumbers are cultivated in the Yellow River irrigation agricultural area in Beiwan Town, Jingyuan County, Baiyin City, Gansu Province, China (104°32′ E, 36°28′ N). The study area has a typical continental climate, with an average annual rainfall of 244 mm. Four treatment groups were investigated: continuous cropping for 4, 8, and 12 years (here after denoted as X4, X8, X12, respectively) and non-continuous cropping soil (in which the crops planted in recent years were mainly wheat and corn) as the control (denoted as Xck). Each treatment is set up with 3 replicates, and the area per replicate is approximately 25.0×9.0 = 225 m^2^. In this study area, the conventional local fertilization method was adopted, and 180.6 kg·ha^-1^ of pig manure, 751.8 kg·ha^-1^ of ammonium dihydrogen phosphate and 268.8 kg·ha^-1^ of potassium sulfate were mainly applied during the growth of cucumber.

### Soil sample collection and processing

On April 23, 2020 (date marking the late stage of cucumber cultivation in solar greenhouses of the study area), rhizosphere soil was sampled. Zero to 5 cm of topsoil was collected, gently shaken to remove loose soil around the roots, brushed off to remove soil attached to the root surface, and was immediately stored in the freezer as rhizosphere samples; three rhizosphere soil samples were taken for each continuous crop period (0, 4, 8, and 12 years) for a total of 12 copies [15].

### Soil chemical analysis

The soil pH value was determined using a HI8314 portable pH meter (Hanna Instruments, Woonsocket, RI, USA) based on a 5:1 soil-water ratio. The soil electrical conductivity (EC) value was measured with a DSJ-308A conductance meter (DSJ - 308A, Jingke, Shanghai, China). The total salt was calculated as follows: total salt (%) = conductance reading value × 5 × K (%) (where, if the conductance reading value ≤ 3, K = 0.064, and if the conductance reading value > 3, K = 0.071). The soil organic matter was determined using potassium dichromate dilution calorimetry. Chemical properties of the soil were determined following Bao’s method [23]. The alkali-hydrolyzed nitrogen content was determined using the alkali-hydrolyzed diffusion method. The content of soil available phosphorus was determined by sodium bicarbonate extraction and molybdenum-antimony resistance colorimetry. The content of soil available potassium was determined by sodium nitrate (NaNO3) extraction and the sodium tetraphenylboron (NaTPB) turbidimetric method [24]. Nitrate nitrogen was determined by dual-wavelength ultraviolet spectrophotometry [25].

### Soil enzyme activity analyses

Soil urease activity was expressed by phenol-sodium hypochlorite colorimetric method in ammoniacal nitrogen (mg·g^-1^) matrix (37°C, 24 h). Catalase activity was determined by potassium permanganate titration and expressed in a 0.1 mol·L^-1^ KMnO_4_ (mL·g^-1^) matrix (25°C, 1 h). Sucrase activity was determined by 3,5-dinitrosalicylic acid colorimetry and expressed in glucose (mg·g^-1^) matrix (37°C, 24 h). The activity of soil alkaline phosphatase was determined by disodium phenyl phosphate calorimetry [26].

### DNA extraction, polymerase chain reaction (PCR) amplification, and sequencing

Soil DNA was extracted using an EZNA® Soil DNA Kit (D4015, Omega, Inc, USA). The extracted genomic DNA was detected by agarose electrophoresis to check the integrity and concentration of genomic DNA, and the DNA was quantified using an ultraviolet spectrophotometer (MMS, Zeiss, Jena, Germany). For bacteria, the V3–V4 region of the 16SrDNA gene was amplified by PCR using the primers (Hanna Instruments, Woonsocket, RI, USA) T341F (5′-CCTACGGGNGGCWGCAG-3′) and 805R (5′-GACTACHVGGGTATCTAATCC-3′) [27]. For fungi, the ITS2 region of the ribosomal RNA gene was amplified by ITS1FI2 (5′-GTGARTCATCGAATCTTTG-3′) and ITS2 (5′-TCCTCCGCTTATTGATATGC-3′) [28]. PCR amplification was performed referring to previous studies [16, 17]. The libraries were sequenced using the NovaSeq PE250 platform [29].

### Data analysis

The samples were sequenced with the Illumina NovaSeq platform. Alpha diversity and beta diversity were calculated using the QIIME2 platform, in which the same number of sequences were extracted randomly by reducing the number of sequences to the minimum of some samples, and the relative abundance (X bacteria or fungi count/total count) was used for bacterial or fungal taxonomy. The Pearson correlation was used to assess the relationships between soil properties and enzyme activities. Microbiological data analyses were conducted in R v. 3.5.2. The sequence alignment of species annotation was performed using the QIIME2 plugin feature classifier, and the alignment databases used were SILVA and UNITE. Redundancy analysis (RDA) was performed using the RDA function in R (version 2.1.3), which was used to study the effect of physical and chemical properties of rhizosphere soil on the composition of rhizosphere soil microbial community.

A one-way analysis of variance (ANOVA) with Duncan’s test was performed using SPSS 26.0 (IBM Corporation, Armonk, NY, USA) to analyze the differences in the soil properties, enzyme activities, and alpha diversity.

## Results

### Effects of cucumber continuous cropping on physicochemical properties and enzyme activities of soil

**Fig 1:**
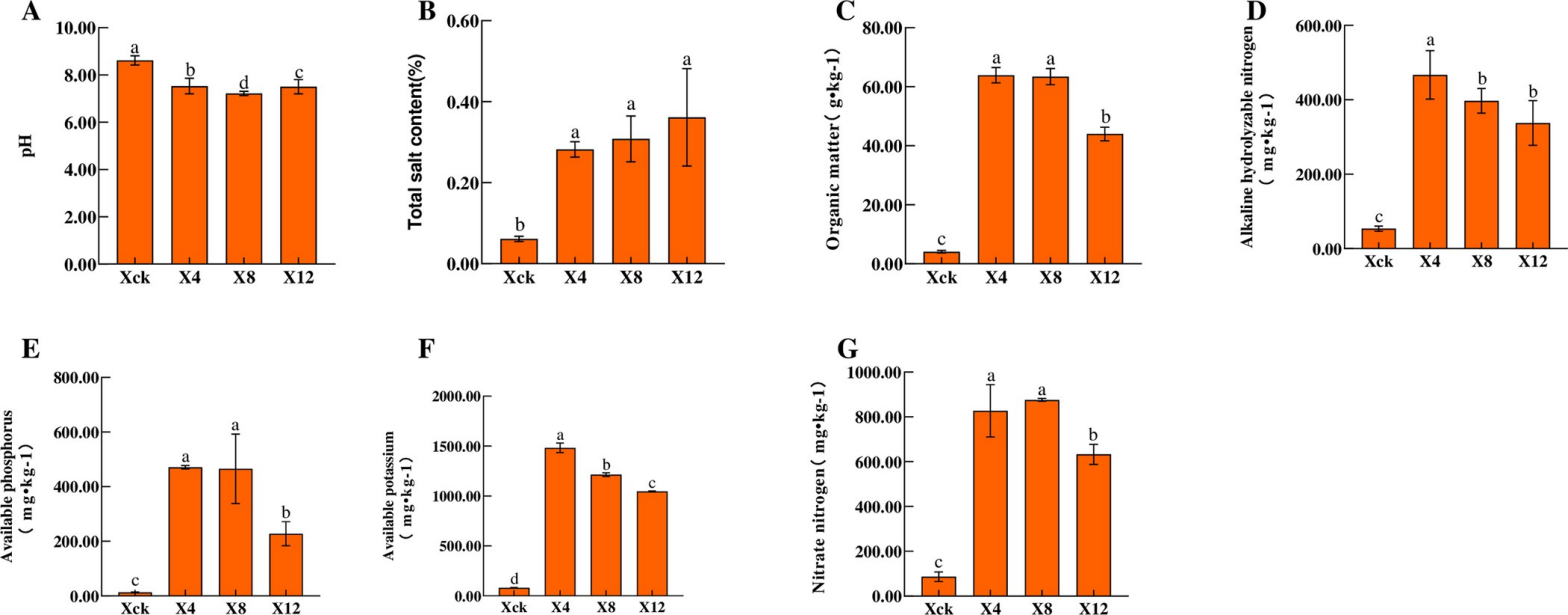
Physicochemical properties of soils under different years of continuous cropping conditions. Xck: non-continuous planting; X4: continuous cucumber cropping for 4 years, X8: 8 years, and X12: 12 years, respectively; (A) pH: acidity, and alkalinity; (B) Total salt content; (C) Organic matter; (D) Alkaline hydrolyzable nitrogen; (E) Available phosphorus; (F) Available potassium; (G) Nitrate nitrogen. Different letters indicate significant differences among the treatments at the *p* < 0.05 significance level. The error bars indicate the standard error of the means (n = 3).

**Fig 2:**
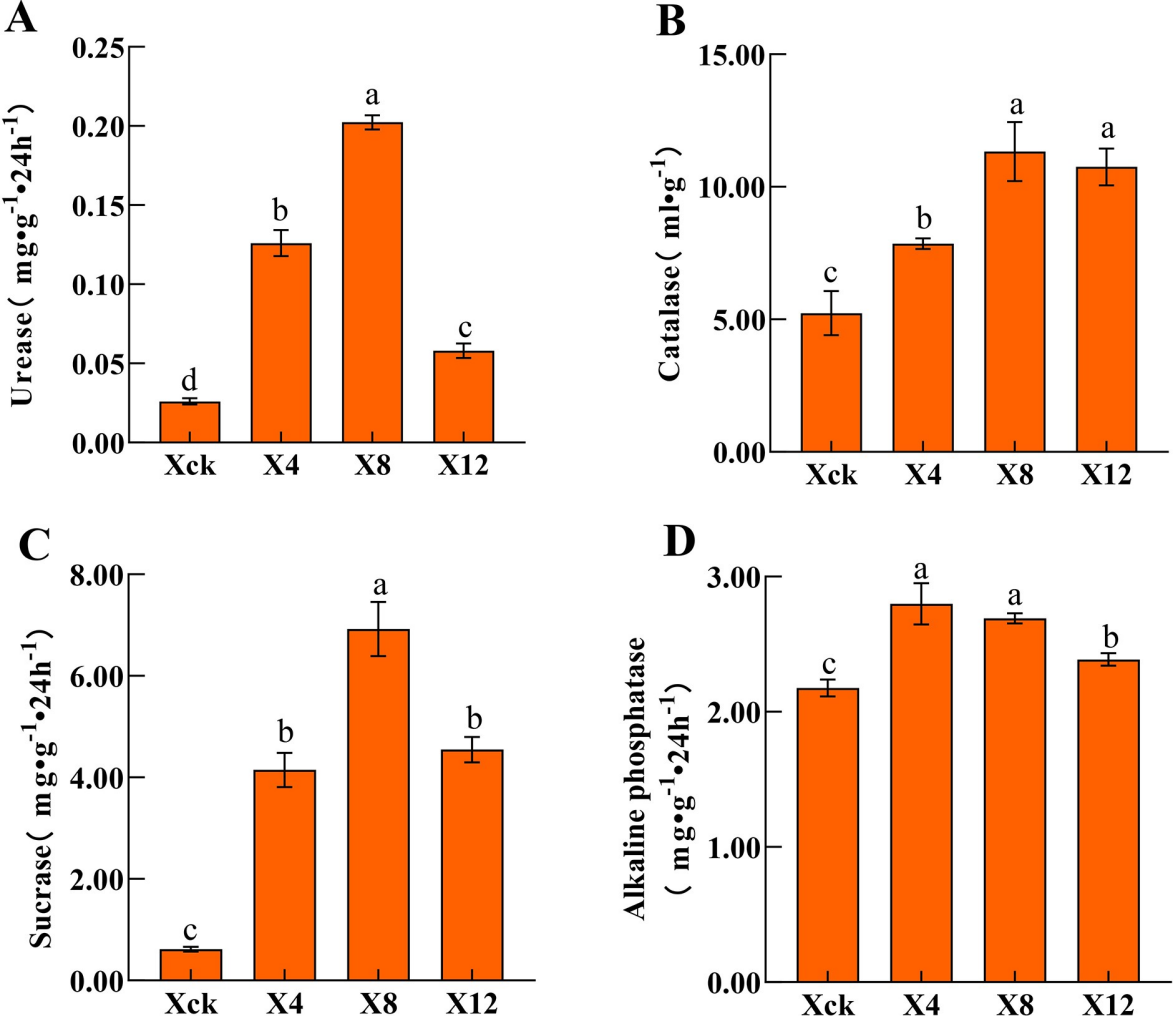
Enzyme activities of soils under different years of continuous cropping conditions. (A) Urease. (B) Catalase. (C) Sucrase. (D) Alkaline phosphatase. Xck: non-continuous planting; X4: continuous cucumber cropping for 4 years, X8: 8 years, and X12: 12 years, respectively. Different letters indicate significant differences among the treatments at the *p* < 0.05 significance level. The error bars indicate the standard error of the means (n = 3).

**Fig 3:**
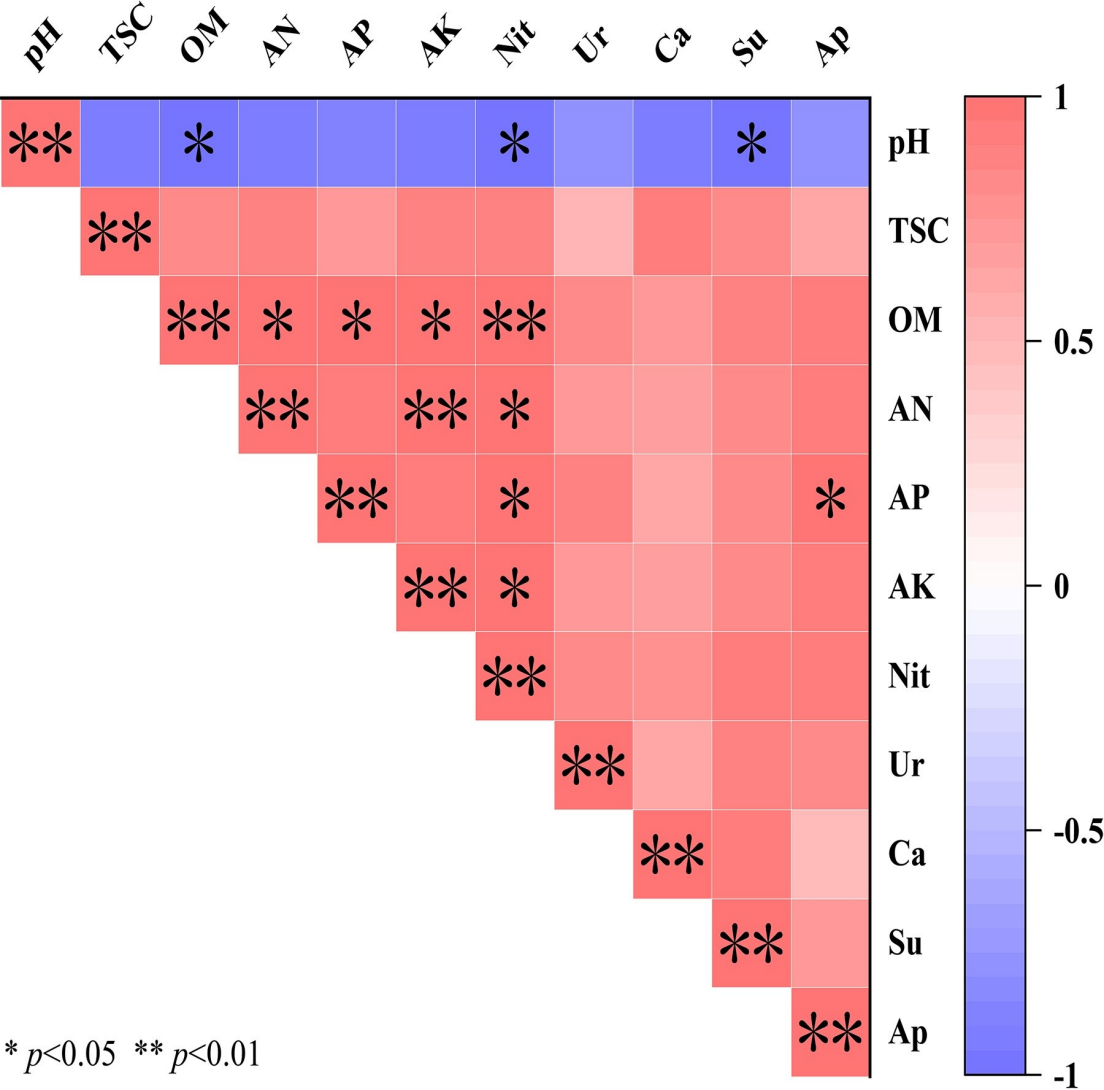
Correlation analysis of soil physicochemical properties and soil enzyme activity of cucumber under different planting years. pH: acidity, and alkalinity; TSC: total salt content; OM: organic matter; AN: alkaline hydrolyzable nitrogen; AP: available phosphorus; AK: available potassium; Nit: Nitrate nitrogen. Ur: Urease; Ca: Catalase; Su: Sucrase; Ap: Alkaline phosphatase.

As shown in Fig 1, with the extension of planting years, the total salt content of the X4, X8, and X12 treatments increased by 360.24%, 402.88%, and 489.11%, while the pH decreased significantly by 12.65%, 16.24%, and 12.95%, respectively, compared to that in Xck. Compared with Xck, the contents of organic matter, alkali-hydrolyzed nitrogen, available phosphorus, available potassium, and nitrate nitrogen increased by 1448.83%, 1438.35%, 966.71%, 768.55%, and 639.14% for X4; 528.24%, 3540.15%, 3496.85%, 1656.61%, and 1728.37% for X8; and 1397.49%, 1189.43%, 852.37%, 908.14%, and 627.95% for X12, respectively. With the increase in continuous cropping years, the physical and chemical properties of soil first increased and then decreased. The pH first decreased significantly and then slowly increased; and the total salt content increased significantly. The organic matter, alkali-hydrolyzed nitrogen, available phosphorus, and available potassium reached their maximum values in the X4 treatment. During the continuous planting of cucumber, the activity of the four soil enzymes tended to first increase and then decrease (Fig 2). The activities of urease, catalase and sucrase peaked after the X8 treatment, reaching 0.202 mg·g^-1^·24 h^-1^, 11.329 ml·g^-1^, and 6.926 mg.g^-1^·24 h^-1^, respectively. The activities of urease, catalase and sucrase increased significantly by 676.58%, 116.34% and 1018.80% in X8 compared with those in Xck, respectively. Moreover, the activity of alkaline phosphatase peaked in X4 (2.799 mg·g^-1^), and then began to decrease. The correlation between the soil physicochemical properties and soil enzyme activities under different planting periods (Fig 3) indicated that pH was negatively correlated with sucrase, and available phosphorus was positively correlated with alkaline phosphatase.

### Soil sample quality testing and different treatment of microbial characteristics

**Fig 4:**
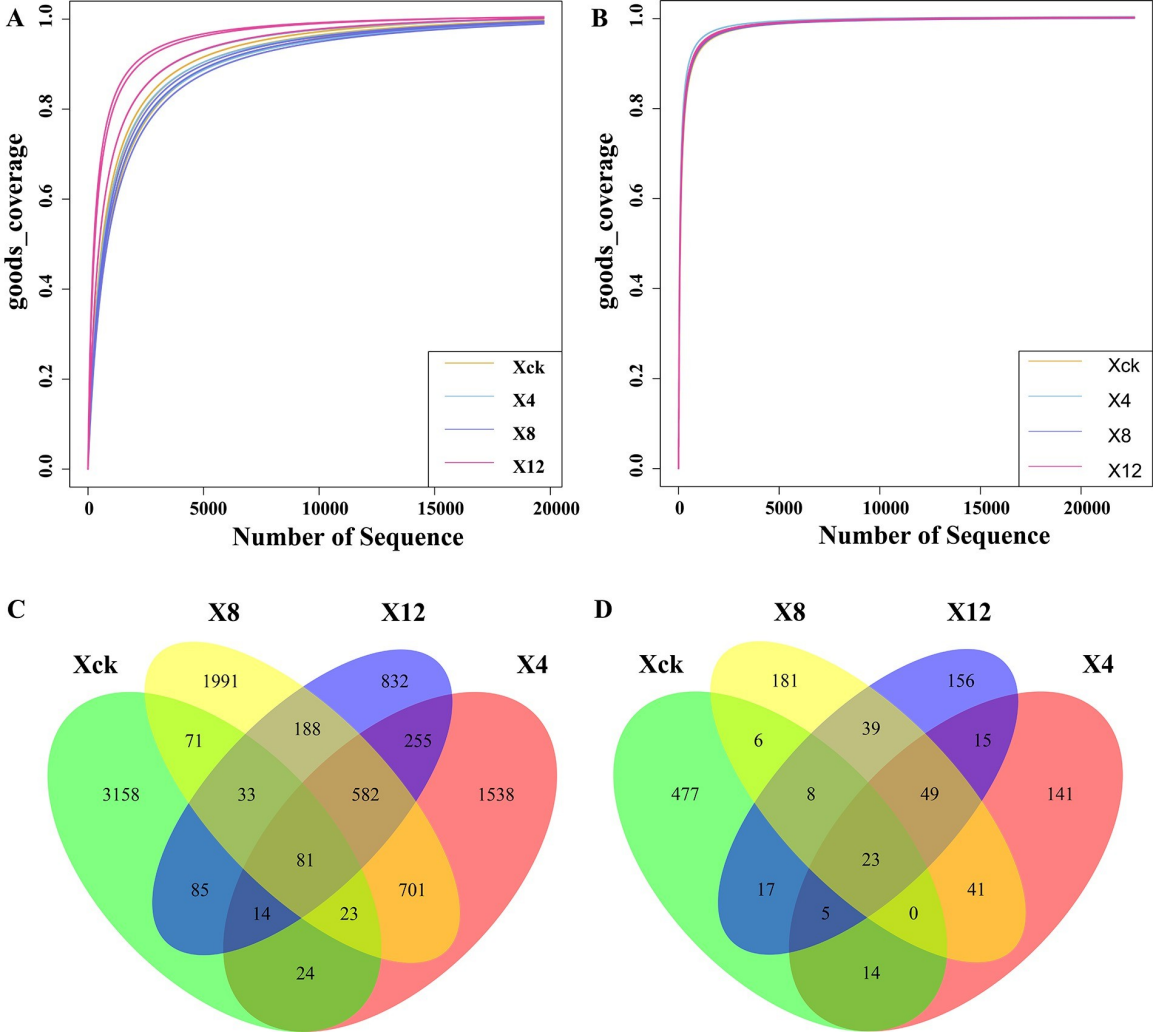
Dilution curves and Venn plots of cucumber rhizosphere soil samples under different continuous cropping periods. Xck: non-continuous planting; X4: continuous cucumber cropping for 4 years, X8: 8 years, and X12: 12 years, respectively.

Good’s coverage index showed that the dilution curve of each sample tended to be relatively flat with an increase in the sample sequencing volume, and the obtained sequence was sufficient to fully capture microbial diversity, indicating that the sequencing data were reasonable and reliable and, met the sequencing requirements, and therefore could accurately reflect the true community of soil samples (Fig 4A and 4B).

The effective sequence with 100% similarity in 12 soil samples was used for feature clustering, and the number of features in each sample was calculated. As shown in Fig 4C and 4D, the total number of bacterial features in X12 was the least which was 2070, 40.67%, 35.67% and 43.60% less than that in Xck, X4 and X8, respectively. After 12 years of continuous cropping, the total number of soil bacteria decreased, and the number of unique species was the lowest, at 832 species lower than Xck (3158), X4 (1538), and X8 (1991). The total numbers of Xck, X4, X8, and X12 fungal features was 550, 288, 306, and 312, respectively. The number of unique species of X4, X8, and X12 in continuous cropping soil was 141, 181, and 156, respectively, which was significantly lower than that of Xck (477). These results indicate that continuous cropping significantly reduces the species and number of microorganisms.

### Alpha diversity index analysis of soil microorganisms

Continuous cropping spans had an effect on the soil microbial diversity and richness indices. Typically, the Chao1 and observed species indices mainly reflect the species richness information of samples, while the Shannon and Simpson indices mainly reflect species richness and evenness. The abundance (observed species and Chao1 indices) and diversity (Shannon and Simpson indices) of the continuously cropped cucumber soil samples are listed in Table 1. The results showed that the species richness and diversity of bacteria and fungi decreased with increasing continuous cropping years. With continuous cucumber planting, the bacterial Shannon, Chao1, and observed species indices decreased overall from 10.15, 1919.84, and 1844 (Xck) to 9.36, 1091.21, and 1085 (X12), respectively. However, there was no significant difference between Xck, X4, and X8. For fungi, the variation trends in the rhizosphere soil richness and diversity indices were basically identical. The richness and diversity indices of Xck were significantly higher than those of the other treatments, but there were no significant differences among the X4, X8, and X12 treatments.

**Table 1:** Comparison of soil bacterial and fungal diversity indices in different continuous cropping years.

	Sample	Shannon Index	Simpson Index	Chao 1 Index	Observed_species
Bacteria	Xck	10.15±0.09a	1±0a	1919.84±136.85a	1844.00±120.67a
	X4	9.75±0.14ab	1±0a	1818.90±211.53a	1747.67±185.67a
	X8	10.08±0.04a	1±0a	2133.39±101.83a	2035.00±84.88a
	X12	9.36±0.2b	1±0a	1091.21±180.17b	1085.00±174.04b
Fungi	Xck	5.74±0.05a	0.93±0.00a	298.07±8.12a	289.33±10.90a
	X4	3.98±0.14b	0.86±0.01b	162.96±36.14b	162.00±35.64b
	X8	4.13±0.04b	0.86±0.00bc	220.65±7.48b	217.67±7.22b
	X12	4.12±0.04b	0.84±0.00c	192.00±3.61b	192.00±3.61b

Xck: non-continuous planting; X4: continuous cucumber cropping for 4 years, X8: 8 years, and X12: 12 years, respectively. values are expressed as mean ± standard deviation (n = 3); different letters indicate a statistically significant difference (*P* < 0.05), the same below.

### Microbial community composition and structure analysis

**Fig 5:**
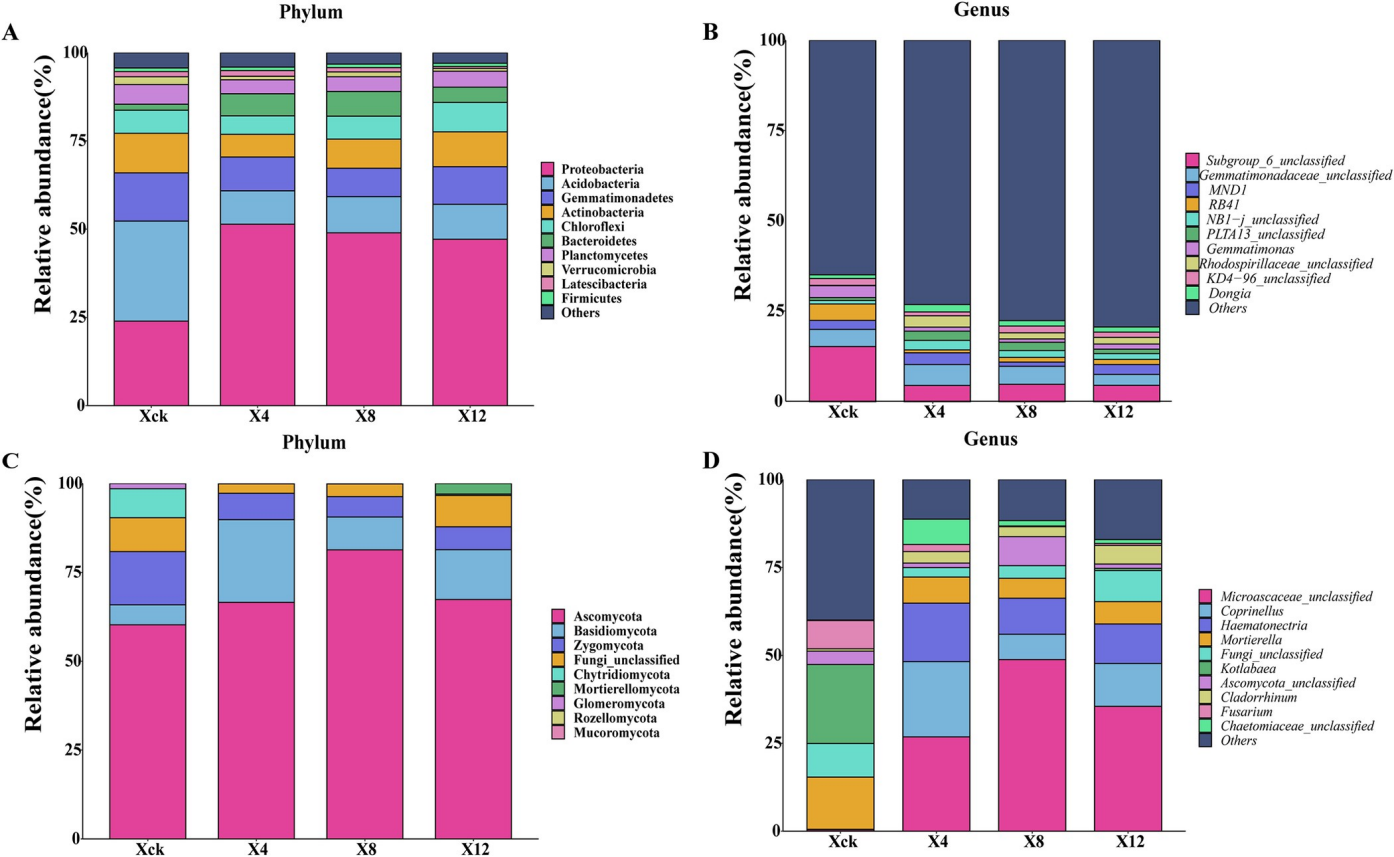
Relative abundance of soil microbial phylum and genus in the rhizosphere of cucumbers for different continuous cropping periods (top 10). Xck: non-continuous planting; X4: continuous cucumber cropping for 4 years, X8: 8 years and X12: 12 years, respectively.

The results of high-throughput sequencing showed that 41 phyla, 133 classes, 860 genera, and 1138 species of bacteria were present in all soil samples. The 10 phylum with the highest abundance were selected to generate a column stack diagram of relative species abundance (Fig 5A). Proteobacteria (42.86%), Acidobacteria (14.49%), Gemmatimonadetes (10.50%), Actinobacteria (8.93%) and green Chloroflexi (6.63%) were the dominant bacteria in the soil (with relative abundance > 5%), accounting for 83.41% of all bacteria. There were no significant differences in Chloroflexi abundance among the different treatments. The relative abundance of Proteobacteria in the Xck treatment was the lowest; while that in X4, X8 and X12 increased by 114.72%, 104.59% and 96.95%, respectively. In addition, the abundance of Acidobacteria, Gemmatimonadetes and Actinobacteria in Xck were significantly higher than those in the other treatments. The relative abundances of the top 10 species at the genus level are shown in Fig 5B. Subgroup_6_unclassified, Gemmatimonadaceae_unclassified, MND1, RB41, NB1-j_unclassified, PLTA13_unclassified, Gemmatimonas, Rhodospirillaceae_unclassified, KD4-96_unclassified, and *Dongia* were the main genera. The top 10 bacteria in relative abundance accounted for 26.37% of all bacteria, among which Xck accounted for the highest proportion (35.19%), X4 and X8 accounted for 26.98% and 22.51%, respectively, and X12 accounted for the lowest proportion (20.79%). Overall, the relative abundance of dominant soil bacteria decreased with an increase in continuous cropping years.

The results showed that there were nine phylum, 30 classes, 214 genera, and 330 species of fungi were present in all soil samples. The overall fungal species in the samples were similar, but the proportion of species richness varied at different taxonomic levels. Ascomycota, Basidiomycota, and Zygomycota were the three main phyla (Fig 5C). The relative abundances of three phylums were the highest in the X4 treatment, reaching 97.26%, while they reached 96.33%, 87.82%, and 80.81% in the X8, X12, and Xck treatments, respectively. The results showed that continuous cropping of cucumbers changed the structure of the soil fungal flora. The relative abundance of the top 10 species at the genus level is shown in Fig 5D. The main genera were: Microascaceae_unclassified, *Coprinellus*, *Haematonectria*, *Mortierella*, Fungi_unclassified, *Kotlabaea*, Ascomycota_unclassified, *Cladorrhinum*, *Fusarium*, and Chaetomiaceae_unclassified. These accounted for 80.02% of all fungi in all treatments, among which X4 accounted for the highest proportion (88.74%); X8 and X12 accounted for 88.33% and 82.95%, respectively; and Xck accounted for the lowest proportion (60.05%). These results indicate that continuous cropping of cucumbers changed the dominant genera of fungi. Overall, continuous cropping changed the structure of the soil microbial community and had a significant impact on the dominant microbial community.

### Soil microbial beta diversity index analysis and heatmapat the genus level under cucumber continuous cropping

**Fig 6:**
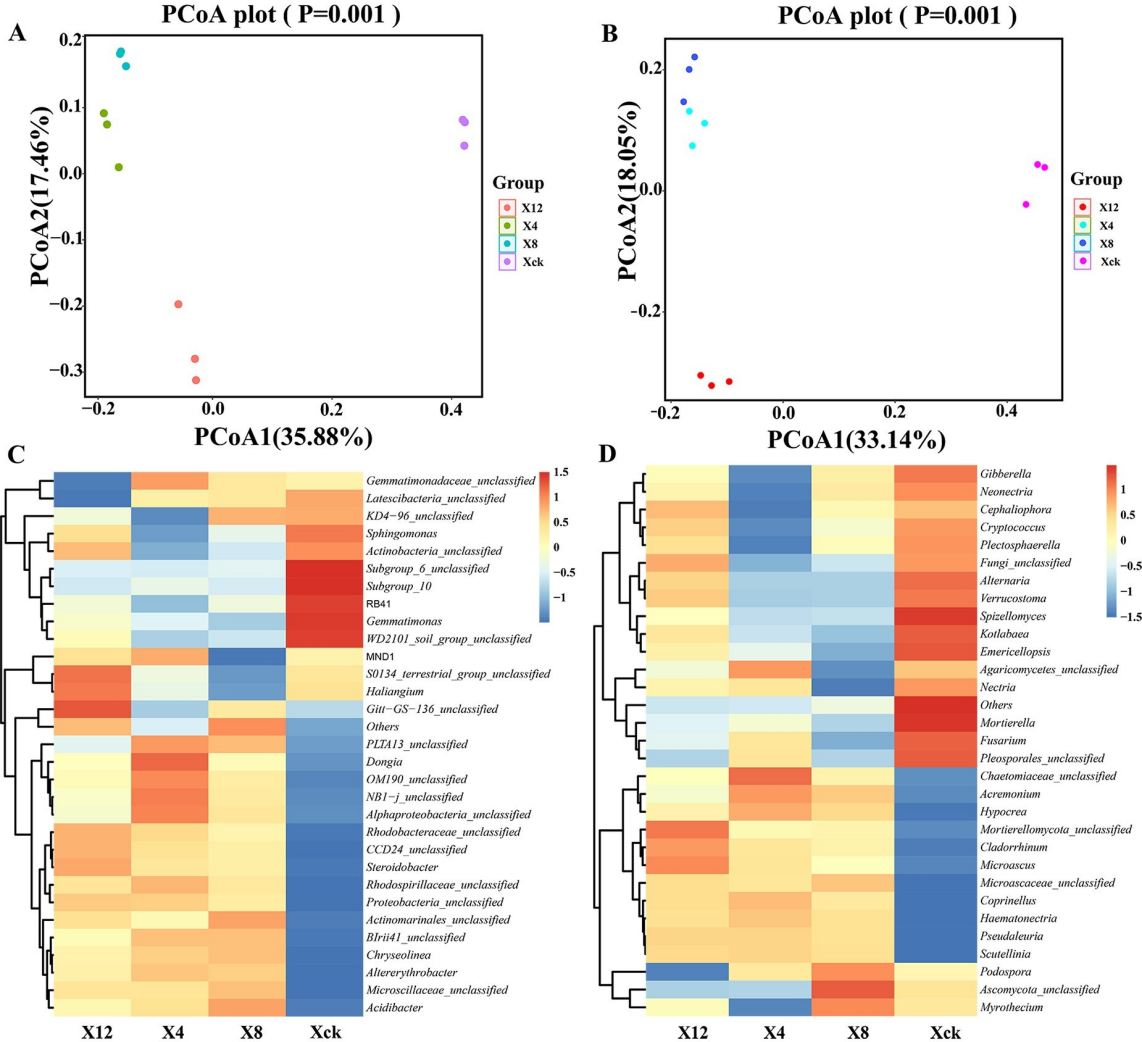
PCoA and genus level heat map analysis of rhizosphere soil microorganisms of cucumber. Xck: non-continuous planting; X4: continuous cucumber cropping for 4 years; X8: 8 years; and X12: 12 years, respectively.

The feature-based weighted UniFrac principal coordinates analysis (PCoA) also showed a difference among cucumber soil samples with different continuous cropping periods. As shown in Fig 6A, PCoA1 and PCoA2 accounted for 35.88% and 17.46% of the total variability in the bacterial data, respectively. Fig 6B shows that PCoA1 and PCoA2 accounted for 33.14% and 18.05% of the total variability in the fungal data, respectively. The distances of the three repeated treatments were similar, indicating that the fungal communities of samples in the same year were highly similar, and the effects of continuous cropping on microbial community structure changed greatly due to the extension of continuous cropping years. PCoA1 clearly separated the microbial communities in the non-continuous cropping soil (Xck) sample series from the other nine samples. Overall, the Xck treatment was distributed on the right, the X4 and X8 treatments were distributed on the upper left, and the X12 treatment was distributed on the lower left, indicating that soil samples of 4 and 8 year continuous cropping had the most similar microbial community compositions. The unweighted UniFrac algorithm showed similar results. However, for clarity, only the weighted Unifrac PCoA graph is presented shown here.

The relative abundance of the microbial community was determined using heat map analysis. According to the similarity comparison, the bacterial community structure in all treatments was divided into four groups (Fig 6C). Treatments X4 and X8 were grouped together and separated from the control Xck and X12. The results show that the community structures of X4 and X8 were the most similar, and the difference was the largest in Xck. Subgroup_6_unclassified, Subgroup_10, RB41, *Gemmatimonas*, and WD2101_soil_group_unclassified were significantly higher in Xck than in X4, X8, and X12.

The heat map of species classification at the soil fungi level for different continuous cucumber cropping planting years is shown in Fig 6D. The soil fungi composition in the different continuous cropping years had certain similarities and differences. Xck had the largest comparison expectation, the darkest color, and the highest species richness, followed in order by X4, X8, and X12. Cluster analysis shows that X8 and X12 had the same abundances of fungal species and similar community structures, and were clustered into one type. The greatest difference was found between Xck and continuous cropping soils (X4, X8, and X12). *Fusarium* and Pleosporales_unclassified were clustered into one category, and the relative abundance in each soil sample was Xck > X4 > X12 > X8; *Kotlabaea* and *Emericellopsis* were clustered into one category, and the relative abundance in each soil sample was Xck > X12 > X4 > X8; *Cladorrhinum* and *Microascus* were clustered into one category, and the relative abundance in each soil sample was X12 > X4 > X8 > Xck; *Acremonium* and *Hypocrea* were clustered into one category, and the relative abundance in each soil sample was X4 > X8 > X12 > Xck.

### RDA between microbial dominant microbial community and soil environmental factors

**Fig 7:**
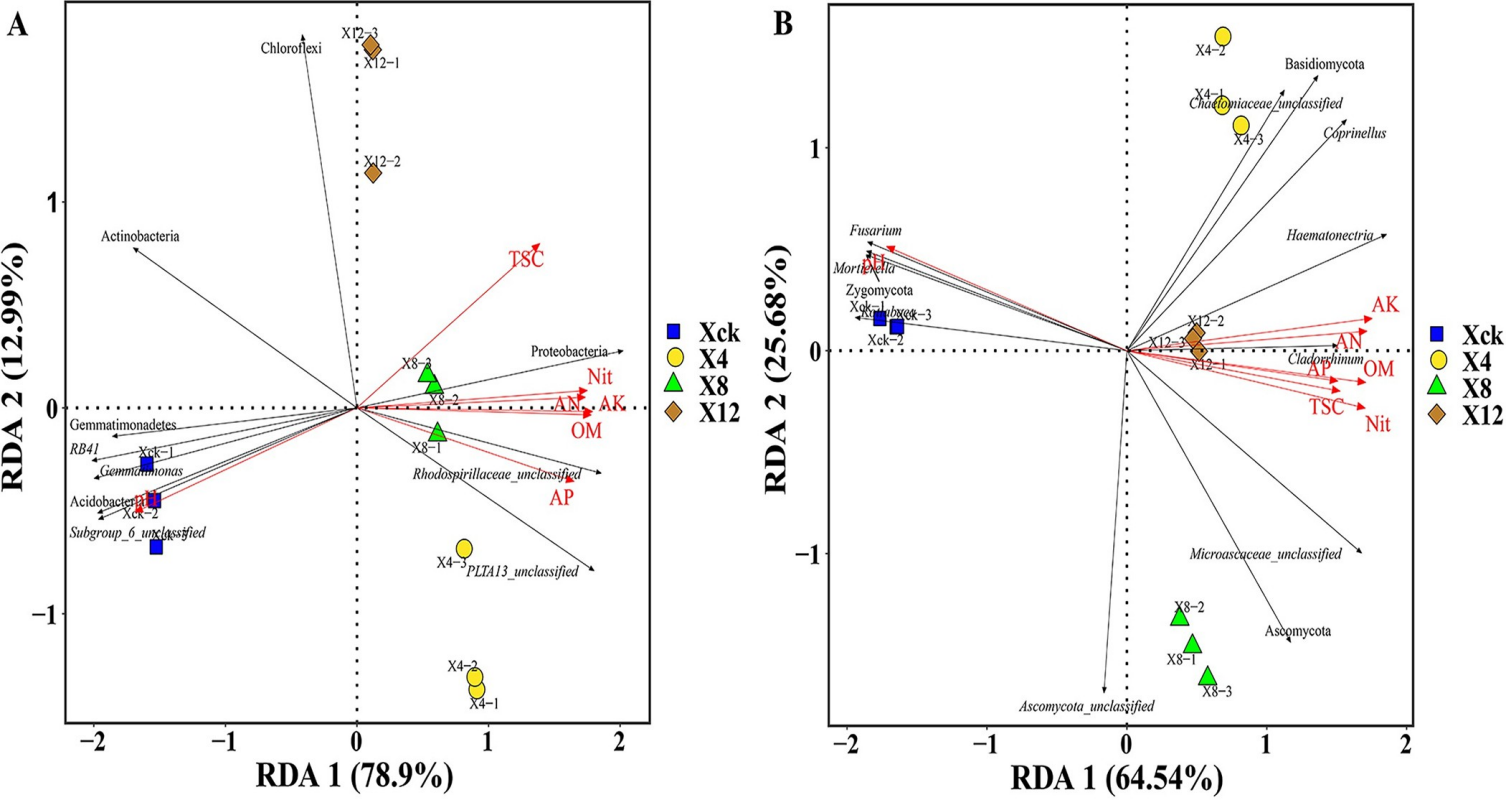
RDA analysis of rhizosphere soil microorganisms and soil physicochemical properties of cucumber. Xck: non-continuous planting; X4: continuous cucumber cropping for 4 years; X8: 8 years, and X12: 12 years, respectively. pH: acidity, and alkalinity; TSC: total salt content; OM: organic matter; AN: alkaline hydrolyzable nitrogen; AP: available phosphorus; AK: available potassium; Nit: Nitrate nitrogen. Each point in the figure represents a sample, The difference between the two points can be explained as: the closer the distance, the higher the similarity of the community structure of the two samples. The arrows represent different influencing factors. When the angle between the influencing factors (between the factor and the sample) is an acute angle, it indicates a positive correlation between the two factors, and when an obtuse angle is negatively correlated, the longer the ray, the greater the effect of the factor.

RDA was conducted for both soil bacterial and fungal communities to elucidate the clustering and separation of samples caused by environmental factors. As shown in Fig 7A, the correlations to axis one and axis two was 78.9% and 12.99%, respectively. This shows that the ranking could better reflect the relationship between the dominant bacterial flora and soil environmental factors. pH was considered the main explanatory factor for the clustering of the bacterial community in Xck. In contrast, the dominant bacterial phylum and total salt content, organic matter, available phosphorus, available potassium, alkaline nitrogen, and nitrate nitrogen were positively correlated in X4 and X8, and the dominant bacterial phylum and total salt content were positively correlated in X12. RDA of fungal communities and environmental factors showed that the correlation to axis one and axis two was 64.54% and 25.68%, respectively. These rankings reflect the relationship between the dominant fungal flora and soil environmental factors (Fig 7B). The pH level, available potassium and alkali-hydrolyzed nitrogen with *Fusarium*, *Mortierella*, *Zygomycota* and *Cladorrhinum* had the smallest angles, which showed a strong correlation; *Fusarium*, *Mortierella*, *Zygomycota*, and *Kotlabaea* were positively correlated with the pH and positively correlated with available potassium, alkali-hydrolyzed nitrogen, available phosphorus, and organic matter. The total salt content was negatively correlated with nitrate nitrogen; Ascomycota, Basidiomycota, Microascaceae_unclassified, *Haematonectria*, Fungi_unclassified, *Cladorrhinum*, and Chaetomiaceae_unclassified were positively correlated with available potassium, alkaline nitrogen, available phosphorus, organic matter, total salt, and nitrate nitrogen, and negatively correlated with pH. Ascomycota_unclassified was negatively correlated with available potassium, alkali-hydrolyzed nitrogen, available phosphorus, organic matter, total salt content, nitrate nitrogen, and pH. In summary, all soil environmental factors all had a measurable impact on the dominant microbial communities of bacteria and fungi, and pH was the main driving factor of the dominant microbial community.

## Discussion

### Changes in the physical and chemical properties in continuous monocropping soil

Long-term and continuous planting of greenhouse vegetables such as cucumber leads to the imbalance of soil physical and chemical properties. Moreover, the excessive accumulation of inorganic ions can lead to soil secondary salinization [30, 31]. In this study, the soil nutrient content under continuous cropping was significantly higher than under non-continuous cropping, mainly due to the unreasonable fertilization and low nutrient utilization in the solar greenhouse [32, 33]; furthermore, the continuous cropping soil also significantly increased the nitrate nitrogen content. With the extension of continuous cropping years, pH significantly decreased in the soil. The main reason for this may be owing to the increase in ions (NO^3–^, SO_4_^2–^, Cl^–^, etc.), which leads to gradual soil acidification [34, 35]. In a previous study, the continuous cropping of cucumber was found to cause cinnamic acid accumulation in the roots [36]. Moreover, in this study, we found that the total salt content continuously increased in the soil with the increase in cultivation years. It may be that the use of low-quality irrigation water has led to the accumulation of soil salt [37].

Soil enzyme activity is an important indicator of soil quality [38]. The activities of urease, catalase, sucrase and alkaline phosphatase in soil under continuous cropping of cucumber first increased and then decreased. The enzyme activities increased after 4 and 8 years of continuous planting but decreased after 12 years (recall Fig 2); this result is similar to the results of the study of Wu et al. [39]. The noted increase of soil enzyme activity under continuous cropping may have been caused by changes in soil properties and microbial diversity [40]. In this study, we found that pH was significantly negatively correlated with sucrase, and available phosphorus was significantly positively correlated with alkaline phosphatase [41] and also increased the accumulation of toxic substances in rhizosphere soil [42]. Such changes can lead to the deterioration of the rhizosphere soil micro-environment, which is not conducive to crop growth [43].

### Effects of soil microbial diversity under cucumber continuous cropping

Soil microbial community composition and diversity are generally considered important soil health indicators [44]; they are critical to the integrity, stability, and sustainability of soil ecosystems [45]. In this study, continuous cropping led to a decrease in the abundance and diversity of bacteria in the rhizosphere soil of cucumbers. This result is similar to the observed trends of soil bacterial diversity and richness under continuous cropping of black pepper [33] and potato [46].

Studies have proven that in poor-quality soils, which tend to contain more fungi, the diversity was low [47]. In this study, compared with the control, the soil fungal diversity was significantly decreased under cucumber continuous cropping in a greenhouse. With the increase in continuous cropping years, the Shannon, Chao1, observation species, and Simpson indices all showed a decreasing trend after an initial increase. Our findings are similar to those of previous studies [13, 40]. With increasing continuous cropping years, the long-term consumption of the soil physical and chemical properties destroyed the soil aggregate structure and micro-ecological environment on which the fungus depends. This is not conducive to soil microbial metabolism, and thus reduces the diversity and uniformity of soil fungi. Such a decrease in microbial diversity and abundance poses a threat to the balance of the soil micro-ecosystem. Therefore, the observed reductions in soil fungal and bacterial diversities of this study were likely related to the loss of certain soil functions [48] such as plant growth promotion and disease inhibition [49, 50], which led to the poor growth of cucumber under continuous cropping conditions.

### Effects of cucumber continuous cropping on the structure of the soil microbial community

Previous studies have shown that continuous cropping changed the composition of the soil microbial community and reduced the community’s relative abundance [51, 52]. At the phylum level in this study, 41 phylum of bacteria were observed, of which Proteobacteria, Acidobacteria, Gemmatimonadetes, Actinobacteria, and Chloroflexi were dominant in the soil (recall Fig 2A). Proteobacteria, Acidobacteria, and Actinobacteria are the most common dominant phylum in different agricultural systems [53]. Both Proteobacteria and Actinomycetes are beneficial soil bacteria that participate in the carbon and nitrogen cycles of various organic matter systems and promote the absorption of soil nitrogen by crops. Anhydride bacteria can degrade plant residues, perform photosynthesis, and participate in the metabolism of carbon compounds. Thus, they play an important role in the circulation of soil materials [54, 55]. In this study, it was found that with increasing continuous cropping years, the phylum Proteobacteria first increased and then decreased. Moreover, the phylum Actinobacteria and Acidobacteria levels both significantly decreased under continuous cropping compared to those under non-continuous cropping. The results are consistent with previous studies [13, 56], confirming that there were fewer actinobacteria in the continuous cropping soil.

As far as fungi are concerned, there are nine phyla. Among them, the three main phyla in the soil samples were Ascomycota, Basidiomycota, and Zygomycota, which is in accordance with the ITS sequencing results of many scholars [57, 58]. Ascomycetes are highly adaptable to the environment and are major decomposers of cellulose, lignin, and organic matter [59]. In this study, there is a trend of first increasing and then decreasing. It can be seen that the decline in the abundance of beneficial soil bacteria may be one of the important reasons for the emergence of continuous cropping disorders [60].

### Influence of soil environmental factors on the microbial community structure

Under continuous cropping conditions, soil physical and chemical properties can well reflect soil health and are known to affect the number and distribution of soil microbial populations. Specifically, nutrient metabolism activities of different vegetation types can lead to certain differences in the physical and chemical properties of soil [61]. A number of studies have shown that environmental factors shaped the structure of microbial communities [61, 62]. In this study, we conducted an RDA of the soil-dominant microbial community of continuous cucumber cropping and soil environmental factors. The results showed that the dominant bacterial and fungal flora were significantly related to soil physical and chemical factors, which is consistent with the results of previous studies showing that soil physical and chemical properties were influenced by the soil microbial community structure [63, 64]. Among them, the bacterial group was significantly positively correlated with soil pH (recall Fig 4A), and a large number of dominant fungal flora was significantly negatively correlated with soil pH (recall Fig 4B). Our results are consistent with those of previous studies showing that soil pH is the most important driving factor affecting soil bacterial and fungal communities [65, 66]. This study found that pH had a significant impact on the soil microbial community, but the dominant microbial flora, such as Acidobacteria, Gemmatimonadetes, *Gemmatimonas*, Actinobacteria, *Kotlabaea*, *Fusarium*, Zygomycota, and *Mortierella*, had different responses to pH. It may be that the appropriate pH range in which different microbes thrive varies [67], which would led to an increase in the abundance of some specific taxa.

## Conclusions

In this study, it was shown that continuous cropping led to acidification of cucumber rhizosphere soil, aggravation of salinization and imbalance of soil nutrients in the Yellow River irrigation area. Moreover, continuous cropping caused a significant increase in the dominant bacterial phylum and fungal phylum in the cucumber rhizosphere soil and a significant decrease in the diversity and abundance of soil bacteria and fungi. RDA further revealed the influence of soil environmental factors on the dominant flora, and pH was found to be the main driving factor of the dominant flora. Therefore, in the production process, attention should be paid to the rational use of chemical fertilizers, and poor-quality irrigation water should be treated before use. At the same time, beneficial microbial agents or crop rotations should be appropriately applied in the single-cropping process to maintain the soil micro-ecological balance and alleviate continuous cropping obstacles.
